# Magnetotransport Studies of Encapsulated Topological Insulator Bi_2_Se_3_ Nanoribbons

**DOI:** 10.3390/nano12050768

**Published:** 2022-02-24

**Authors:** Gunta Kunakova, Edijs Kauranens, Kiryl Niherysh, Mikhael Bechelany, Krisjanis Smits, Gatis Mozolevskis, Thilo Bauch, Floriana Lombardi, Donats Erts

**Affiliations:** 1 Institute of Chemical Physics, University of Latvia, 19 Raina Blvd., LV-1586 Riga, Latvia; edijs.kauranens@lu.lv (E.K.); kiryl.niherysh@lu.lv (K.N.); donats.erts@lu.lv (D.E.); 2 Research and Development Department, Integrated Micro- and Nanosystems, Belarusian State University of Informatics and Radioelectronics, P. Brovki Str. 6, 220013 Minsk, Belarus; 3 Institut Européen des Membranes, IEM, UMR 5635, University of Montpellier, ENSCM, CNRS, 34095 Montpellier, France; mikhael.bechelany@umontpellier.fr; 4 Institute of Solid State Physics, University of Latvia, Kengaraga 8, LV-1063 Riga, Latvia; smits@cfi.lu.lv (K.S.); gatis.mozolevskis@cfi.lu.lv (G.M.); 5 Quantum Device Physics Laboratory, Department of Microtechnology and Nanoscience, Chalmers University of Technology, SE-41296 Goteborg, Sweden; thilo.bauch@chalmers.se (T.B.); floriana.lombardi@chalmers.se (F.L.)

**Keywords:** Bi_2_Se_3_ nanoribbons, ZnO, magnetotransport

## Abstract

The majority of proposed exotic applications employing 3D topological insulators require high-quality materials with reduced dimensions. Catalyst-free, PVD-grown Bi_2_Se_3_ nanoribbons are particularly promising for these applications due to the extraordinarily high mobility of their surface Dirac states, and low bulk carrier densities. However, these materials are prone to the formation of surface accumulation layers; therefore, the implementation of surface encapsulation layers and the choice of appropriate dielectrics for building gate-tunable devices are important. In this work, all-around ZnO-encapsulated nanoribbons are investigated. Gate-dependent magnetotransport measurements show improved charge transport characteristics as reduced nanoribbon/substrate interface carrier densities compared to the values obtained for the as-grown nanoribbons on SiO_2_ substrates.

## 1. Introduction

Three-dimensional topological insulators (3D-TIs) are among the major materials in the class of topological materials. 3D-TIs have attracted significant research interest due to their unusual surface properties. Carriers originating from topological surface states exhibit a Dirac cone in the band structure [1] and charge transport via these states is protected against backscattering from non-magnetic impurities [2,3]. If proximitized with an s-wave superconductor, superconductivity induced in the topological surface states is unconventional and predicted to host Majorana fermions [4,5]. The exploitation of these exotic surface properties is advantageous for a variety of applications, for example, in topological quantum computing [6], spintronics [7,8], and in the development of new-concept electronic devices [9]. The surface states of TIs are metallic while the bulk of the material, which is expected to be an insulator, is highly doped due to the formation of native defects [10]. This aspect remains the main challenge in accessing the surface-state charge transport, hampering progress towards the development of applications beyond fundamental studies.

Owing to their large surface-to-volume ratio, 3D-TI nanowires and nanoribbons are promising candidates with which to achieve truly topological surface-state-dominated charge transport without any contribution from the bulk. Their nanosized geometry provides even more functionalities because of the low number of transport modes [11], which is particularly important for probing Majorana states [6]. Remarkable improvements in material quality have been demonstrated in 3D-TIs doped with native-defect-compensating substitutions. Nearly insulating bulk with a charge carrier density of ~10^15^ cm^−3^ has been reported in single crystals of BiSbTeSe_2_ [12] (BSTS), but this approach is not fully successful in nanowires and nanoribbons. Here, precise and reproducible concentrations of dopants are challenging to obtain, and they are achieved at the expense of charge carrier mobility [13].

Nanoribbons of Bi_2_Se_3_ have been reported to be nearly ideal 3D-TIs, practically without any bulk conduction, and with exceptionally high carrier mobilities [14,15]. However, Bi_2_Se_3_ is prone to the formation of surface accumulation layers [16]; this is particularly evident in thin nanoribbons, where the thickness is comparable with the Debye screening length [14,17,18]. The majority of the proposed 3D-TI-nanoribbon-based electronic devices require good tunability of their chemical potential for accessing surface Dirac carriers in a controlled manner. This can be achieved by employing electrostatic gating techniques. However, additional trivial carriers with large densities form at the nanoribbon surfaces, or at the interface with the substrate, which cannot be effectively depleted by common electrostatic gating techniques. Therefore, more effort is needed to prevent the uncontrolled formation of surface accumulation layers in Bi_2_Se_3_ nanoribbons.

The use of surface-capping layers for Bi_2_Se_3_ and Bi_2_Te_3_ has proven to be beneficial to protect against environmental doping [19] and to probe surface state transport. Widely used capping layer materials are Te or Se, and the oxide layers of ZnO or Al_2_O_3_ [13,20,21], deposited on the top surface of the material. This allows more efficient electrostatic tuning of the Fermi level [21], while in the case of Bi_2_Se_3_ nanoribbons, where the accumulation layer is formed at the nanoribbon/substrate interface [14,17], other approaches have to be considered.

In this work, we used atomic layer deposition (ALD) to fabricate all-around ZnO-capped or -encapsulated Bi_2_Se_3_ nanoribbons. The choice of selecting ZnO as an encapsulation layer material was based on the fact that thin layers of high-quality ZnO are possible to grow at moderate temperatures. This is particularly important for preserving the stoichiometry of Bi_2_Se_3_, as elevated temperatures may cause the unwanted out-diffusion of Se, which increases the doping of the bulk. Comparative magnetotransport studies of individual encapsulated and as-grown Bi_2_Se_3_ nanoribbons from the same batch synthesis show that the encapsulation layer of ZnO helps to minimize the impact of the accumulation layer at the nanoribbon/substrate interface and improves the tunability of the chemical potential using a back-gate. These findings are important for the implementation of 3D-TI-nanoribbon-based topological quantum devices.

## 2. Materials and Methods

Free-standing Bi_2_Se_3_ nanoribbons were grown on glass substrates using catalyst-free physical vapor deposition (PVD). The growth procedure is described in detail elsewhere [22]. As-grown nanoribbons were mechanically transferred to prepatterned Si/300 nm SiO_2_ chips by bringing the chip and the glass substrate into contact with each other. The glass substrate with the remaining free-standing nanoribbons was then covered with 2 nm of ZnO, using ALD at ~100 °C, in a home-built set-up.

Flakes of hexagonal boron nitride (h-BN) were exfoliated from h-BN single crystals (2D semiconductors) and transferred to prepatterned Si/300 nm SiO_2_ chips. ZnO-encapsulated Bi_2_Se_3_ nanoribbons were then transferred to the chips partially covered with thin flakes of h-BN. Standard electron beam lithography processing was used to define electrical contacts to individual Bi_2_Se_3_ and ZnO/Bi_2_Se_3_ nanoribbons. After developing the resist, the samples were etched for 60 s in H_2_O/HCl/H_2_O_2_/CH_3_COOH solution [23] at room temperature to remove the surface oxide layer, and layers of Ti (3 nm) and Au (80 nm) were evaporated shortly after the etching to ensure formation of ohmic contacts.

Charge transport measurements were conducted in a Physical Property Measurement System (PPMS) Dynacool, equipped with a 9 T magnet, at a base temperature of 2 K. In magnetoresistance measurements, a magnetic field *B* was applied perpendicularly to the nanoribbon surface. Electrode pair I^+^/I^−^ (see Figure 2a) was used as the current electrodes to ensure a uniform flow of current in the nanoribbon, while the remaining electrodes V_1_ to V_8_ were employed as the voltage probes. Longitudinal resistance R_xx_ was recorded using, for example, electrode pair V_3_/V_7_ while the transversal resistance R_xy_ was measured across the pair V_5_/V_6_. For this particular nanoribbon device, voltage electrodes V_1_ to V_4_ are positioned where the nanoribbon is on top of the h-BN flake (~30 nm in thickness), while the other voltage electrodes are located on the nanoribbon part, which is in direct contact with the SiO_2_.

In order to determine whether the ZnO had covered the free standing Bi_2_Se_3_ nanoribbons, the nanoribbons were transferred to Cu grids and imaged through high-resolution transmission electron microscope (HR-TEM Technai, Fei, Eindhoven, Netherland).

## 3. Results and Discussion

**Figure 1:**
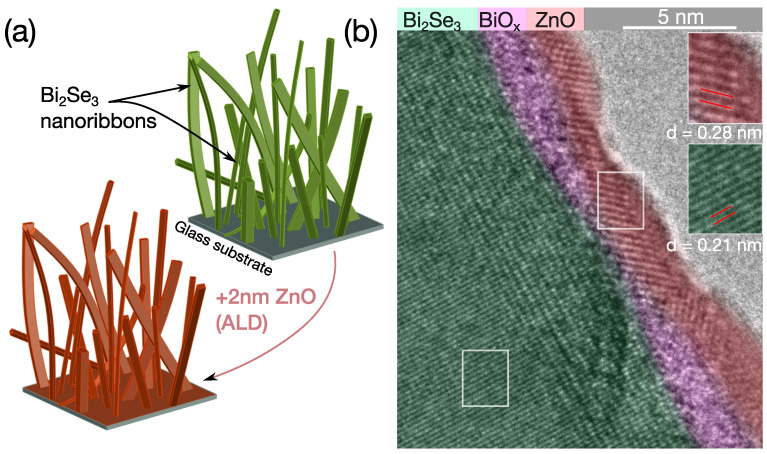
(**a**) Schematic representation of catalyst-free PVD-synthesized free-standing Bi_2_Se_3_ nanoribbons on glass substrate; (**b**) false-colored HR-TEM image of a Bi_2_Se_3_ nanoribbon after encapsulation with a thin layer of ZnO.

Simplified schematics illustrating the free-standing nanoribbons and encapsulation with a thin ZnO layer are shown in Figure 1a. The HR-TEM studies of the ZnO/Bi_2_Se_3_ nanoribbons reveal a crystalline layer, with a thickness of ~2 nm, at the nanoribbon surfaces. In total, five different nanoribbons of various geometries were examined, and a crystalline surface layer was formed in all of them. The *d*-spacing value estimated from the lattice fringes of Bi_2_Se_3_ is 0.21 nm, which is in good agreement with the previous studies [22]. The *d*-spacing value determined for the ZnO of 0.28 nm corresponds to (100) planes of hexagonal wurtzite [24]. The interface between the Bi_2_Se_3_ and ZnO is separated by a layer of amorphous material, with a thickness of ~1.5–2 nm. This layer corresponds to native oxide of Bi_2_Se_3_, BiO_x_ (see Figure 1b), which is always present on surfaces of Bi_2_Se_3_ [19].

**Figure 2:**
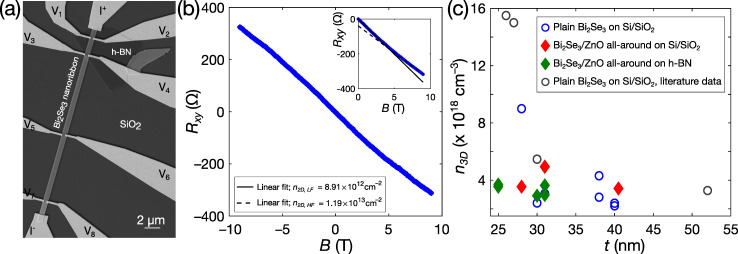
(**a**) SEM image of a Bi_2_Se_3_ nanoribbon Hall-bar device; (**b**) Hall resistance $R_{xy}\left(B\right)$ for the ZnO/Bi_2_Se_3_ nanoribbon device A3t (see Table S1), measured at back-gate voltage $V_{g}$ = 0 V. The inset shows anti-symmetrized $R_{xy}\left(B\right)$ data with linear fit in the 0–2.5 T range (black solid curve), and in the 7–9 T range (black dashed curve); (**c**) Hall carrier density of Bi_2_Se_3_ and ZnO/Bi_2_Se_3_ nanoribbons, plotted versus the nanoribbon thickness. In the case of the ZnO/Bi_2_Se_3_ nanoribbons, total thickness *t* is reduced by 4 nm, accounting for the two ~2 nm thick ZnO layers. Gray data points correspond to the data from [14]; here, the carrier density is calculated from the same magnetic field range (0–2.5 T).

One of the fabricated nanoribbon Hall-bar devices used in the magnetotransport measurements is depicted in Figure 2a. The measured $R_{xy}\left(B\right)$ at zero back-gate voltage is shown in Figure 2b. In order to minimize the error from misaligned electrodes, the data were anti-symmetrized as a function of the magnetic field (see inset of Figure 2b). The $R_{xy}\left(B\right)$ dependences for all the measured nanoribbons were nonlinear. The absolute value of the slope calculated from the high magnetic field range 7–9 T was always smaller than the value determined from the 0–2.5 T range. This nonlinearity points to the charge carriers originating from two or more carrier bands characterised by different densities/mobilities. The initial carrier density $n_{3D}$ can be calculated from the low or high magnetic field slope of $R_{xy}\left(B\right)$ as:

(1) $$\frac{1}{n_{3D}e}=t\frac{dR_{xy}}{dB}\times \frac{w}{w_{h}}.$$

Here, t is the nanoribbon thickness, w is the nanoribbon width, $w_{h}$ is the distance between the Hall contacts, and e is the elementary charge. The calculated values for the 2D carrier densities ($n_{2D}=n_{3D}\cdot t$) from both the 0–2.5 T and 7–9 T regions for the as-grown and ZnO-encapsulated nanoribbons from the same batch synthesis are listed in Table S1 (see Supplementary Information (SI)). The values estimated from the 7–9 T range are about 20–30% higher than those obtained from the 0–2.5 T range.

Figure 2c shows the $n_{3D}$ of the as-grown Bi_2_Se_3_ and ZnO/Bi_2_Se_3_ nanoribbons, plotted as a function of the nanoribbon thickness. The data correspond to the values calculated from the 0–2.5 T range, since in high magnetic fields, some nanoribbons showed the presence of Shubnikov–de Haas oscillations in $R_{xx}\left(B\right)$, additionally impacting the $R_{xy}\left(B\right)$ dependence.

The charge carrier density $n_{3D}$ for the as-grown Bi_2_Se_3_ nanoribbons with thicknesses of ~30–40 nm is about ~3.5 × 10^18^ cm^−3^, and it increases to ~9 × 10^18^ cm^−3^ for the 28-nanometer-thin nanoribbon. This peculiar $n_{3D}\left(t\right)$ dependence of the catalyst-free PVD-grown Bi_2_Se_3_ nanoribbons has been reported previously [14]. The increased 3D charge carrier density for nanoribbons of thicknesses below ~30 nm is due to the accumulation layer of a large carrier density of ~1.3 × 10^13^ cm^−2^ (see Table 1), formed at the nanoribbon’s bottom surface/substrate interface [14]. Figure 2c also includes the values of the carrier densities reported in [14] (gray points). In this work, the obtained $n_{3D}\left(t\right)$ for the as-grown ribbons is similar to those previously reported in the literature.

The $n_{3D}$ values for the ZnO-encapsulated Bi_2_Se_3_ nanoribbons are close to those determined for the as-grown nanoribbons with thicknesses of ~30–40 nm, and are also about ~3.5 × 10^18^ cm^−3^. A pronounced increase of $n_{3D}$ of the thin ZnO-encapsulated nanoribbons (*t* < 30 nm) is not observed, indicating that the overall carrier density in the accumulation layer could be smaller compared to the as-grown Bi_2_Se_3_ nanoribbons.

**Figure 3:**
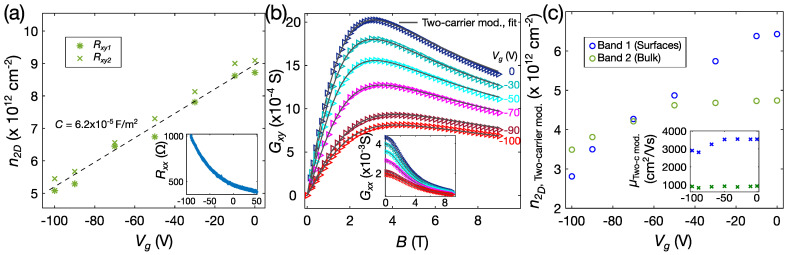
(**a**) Charge carrier density $n_{2D}(=n_{3D}t)$ as a function of the back-gate voltage $V_{g}$. Here, $n_{2D}$ is calculated from the anti-symmetrized $R_{xy}\left(B\right)$ data in the 0–2.5 T range. $R_{xy1}$ and $R_{xy2}$ represent the Hall resistances measured using two different pairs of transversal electrodes, on the same nanoribbon. Black dashed line is the linear fit, and the capacitance estimated from the slope is 6.2 × 10^−5^ F/m^2^. In the inset—longitudinal resistance $R_{xx}$ as a function of the $V_{g}$; (**b**) conductance tensor element $G_{xy}\left(B\right)$ at different applied $V_{g}$, fitted with the two-carrier model, inset shows fitted $G_{xx}\left(B\right)$ curves; (**c**) from the two-carrier model extracted parameters of the two bands: carrier densities $n_{1}$; $n_{2}$, and mobilities $\mu_{1}$; $\mu_{2}$ (in the inset) versus the back-gate voltage. All the data shown correspond to the ZnO/Bi_2_Se_3_ nanoribbon A3t.

The charge carrier density $n_{2D}$ as a function of the back-gate voltage $V_{g}$ for a 28-nanometer-thin ZnO-encapsulated Bi_2_Se_3_ nanoribbon on h-BN is plotted in Figure 3a. The applied back-gate voltage directly affects the nanoribbon bottom surface/substrate interface, and at higher $V_{g}$ values, some parts of the nanoribbon bulk as well. The slope of the $n_{2D}(V_{g})$ gives an indication of the capacitance of this field-effect device, and *C* ≈ 6.2 × 10^−5^ F/m^2^. In order to effectively deplete the majority of the initial carriers of ~9 × 10^12^ cm^−2^, one would need to apply approximately twice as high a voltage to the back-gate, which is not feasible for this device. Nevertheless, the $n_{2D}(V_{g})$ data are helpful for the study of the properties of the nanoribbon/substrate interface. The $R_{xx}\left(V_{g}\right)$ data of the same ribbon reflect the $n_{2D}(V_{g})$ characteristics (see inset of Figure 3a). The absence of maxima or saturation in the $R_{xx}\left(V_{g}\right)$ indicates that the Fermi energy $E_{F}$ remained above the Dirac point in the entire measured $V_{g}$ range. To tune the $E_{F}$ to the Dirac point, which is important for accessing the charge carriers exclusively from the surface Dirac states, ultra-thin (*t* ~ 10 nm) Bi_2_Se_3_ nanoribbons would be needed. Another aspect for improving the gate tunability is the thickness and permittivity of the gate dielectric, i.e., a thinner dielectric layer than the 32 nm of h-BN on 300 nm of SiO_2_ could be used (ε~3–4), or, alternatively, one could choose a SrTiO_3_ substrate, in which the relative dielectric constant at low temperatures is in the order of 10^3^–10^4^.

Since the $R_{xy}\left(B\right)$ curves clearly indicate the presence of charge carriers from several carrier bands, we analysed the magnetotransport data using the two-carrier model.

Here, the conductance tensor elements $G_{xy}$ and $G_{xx}$ as a function of the magnetic field can be written as [25,26]:

(2a) $$G_{xy}\left(B\right)=eB\left(\frac{n_{1}\mu_{1}^{2}}{1+\mu_{1}^{2}B^{2}}+\frac{n_{2}\mu_{2}^{2}}{1+\mu_{2}^{2}B^{2}}\right)$$

(2b) $$G_{xx}\left(B\right)=e\left(\frac{n_{1}\mu_{1}}{1+\mu_{1}^{2}B^{2}}+\frac{n_{2}\mu_{2}}{1+\mu_{2}^{2}B^{2}}\right)$$

with parameters $n_{1},n_{2}$ and $\mu_{1},\mu_{2}$ representing the carrier densities and mobilities of the two bands, respectively. $G_{xy}$ and $G_{xx}$ from the measured resistances are calculated as:

(3a) $$G_{xy}\left(B\right)=-\frac{R_{xy}^{\prime }}{{R^{\prime }}_{xy}^{2}+{R^{\prime }}_{xx}^{2}}$$

(3b) $$G_{xx}\left(B\right)=-\frac{R_{xx}^{\prime }}{{R^{\prime }}_{xy}^{2}+{R^{\prime }}_{xx}^{2}}$$

$R_{xy}^{\prime }$ is the Hall resistance, corrected considering the geometry of a nanoribbon Hall-bar device, and is equal to $R_{xy}\;w/w_{c}$. $R_{xx}^{\prime }$ is the sheet resistance, equal to $R_{xx}\;w/L$. The calculated conductance tensor elements as a function of magnetic field for different applied back-gate voltages are fitted with Equations (2a) and (2b) and plotted in Figure 3b. For the nanoribbon A3t, the extracted value of the charge carrier density of band 1 is $n_{1}=$ 6.43 × 10^12^ cm^−2^ and the mobility $\mu_{1}=$ 3530 cm^2^/Vs, while the carrier density and mobility of band 2 are $n_{2}=$ 4.74 × 10^12^ cm^−2^ and $\mu_{2}=$ 990 cm^2^/Vs, respectively. These parameters of the two bands are similar to those estimated for other ZnO-encapsulated Bi_2_Se_3_ nanoribbons (see Table 1).

The extracted carrier density values $n_{1}$ and $n_{2}$ of the two bands change with the applied back-gate voltage. The value $n_{1}$ scales linearly with the applied back-gate voltage and is reduced by ~50% at $V_{g}$ = −100 V. Instead, $n_{2}$ is practically insensitive to $V_{g}$ in the 0–−50 V range, while at $V_{g}$ > −50, V starts to decrease more rapidly.

In what follows, we discuss a possible scenario that would account for this behaviour. Band 1 is affected by the back-gate voltage much more strongly; therefore, the carrier density $n_{1}$ can most likely be associated with the surface states. As the nanoribbons are fully encapsulated by the ZnO protection layer, the mobilities of the nanoribbon top and bottom surfaces can be expected to have similar values, and carriers from both surfaces would appear in the same channel ($n_{1}$) of the two-band model. The bulk mobilities are typically reported to be of much lower values [27], and the $\mu_{1}$ of 3530 cm^2^/Vs is more than three times larger than the value of $\mu_{2}$. For nanoribbon A1b, where the $\mu_{1}$ is 4700 cm^2^/Vs, SdH oscillations with two dominating frequencies are observed (see Figure S2, SI). One of the frequencies of ~99 T is similar to that observed in the catalyst-free PVD-grown Bi_2_Se_3_ nanoribbons, which have previously been reported to represent the surface Dirac states from the nanoribbon top surfaces [14,22,28]. This gives the carrier density of the nanoribbon top surface of $n_{TS\;SdH}$~2.4 × 10^12^ cm^−2^. The carriers from the top surface are most likely insensitive to the back-gate voltage, as the nanoribbon is of a relatively large thickness. The bottom surface/interface $n_{BS,\;Int.}$ carrier density at $V_{g}$ = 0 V would be then $n_{1}-n_{TS\;SdH}$ ≈ 4 × 10^12^ cm^−2^, which would not be very different from all the ZnO/Bi_2_Se_3_ nanoribbons transferred onto the h-BN (4.03, 3.84 and 4.78 × 10^12^ cm^−2^ for the nanoribbons A3t, D3b, and A1b, respectively). These low values corroborate that the ZnO encapsulation of Bi_2_Se_3_ nanoribbons mitigates the creation of an accumulation layer.

Band 2 with carrier density $n_{2}$ can be assumed to correspond to the bulk carriers. Above −50 V, when the bottom surface/interface carriers are partly depleted, a fraction of the bulk carriers also starts to be affected by the back-gate voltage, and at $V_{g}$ = −100 V, the $n_{2}$ is reduced to ~3.5 × 10^12^ cm^−2^. At $V_{g}$ = 0 V, the $n_{2}$ is 4.74–5.31 × 10^12^ cm^−2^ (see Table 1), and if rescaling to the 3D values: 1.46–1.64 × 10^18^ cm^−3^. Peculiarly enough, the second frequency of the aforementioned SdH oscillations of the nanoribbon A1b (Figure S2, SI), with the highest $\mu_{2}$, gives 1.44 × 10^18^ cm^−3^. This value is close to the 3D bulk carrier densities determined from band 2.

## 4. Conclusions

To conclude, the application of a ZnO encapsulation layer to topological insulator Bi_2_Se_3_ nanoribbons and the use of h-BN as a substrate help to improve the nanoribbon/substrate interface properties. Thin layers of crystalline ZnO have no degrading impact on the overall transport characteristics of Bi_2_Se_3_ nanoribbons. The 3D charge carrier densities for nanoribbons of different thicknesses are of the same order as the values determined for as-grown nanoribbons with thicknesses of 30–40 nm. The reduced surface carrier density extracted from two-band Hall analysis points towards a reduction in the interface accumulation layer when encapsulating Bi_2_Se_3_ nanoribbons with a thin ZnO layer. Moreover, the ZnO-encapsulated nanoribbons show excellent Hall mobility. The presence of the Shubnikov–de Haas oscillations confirms that the high quality of catalyst-free PVD-grown Bi_2_Se_3_ nanoribbons stays preserved if ZnO is used as an encapsulation layer. This approach of all-around encapsulation in combination with ultra-thin Bi_2_Se_3_ nanoribbons, transferred to mono or few layer h-BN substrates, would be beneficial to controllably achieve ambipolar transport in Bi_2_Se_3_.

## Figures and Tables

**Table 1:** Summary of the carrier densities (cm^−2^) and mobilities (cm^2^/Vs) extracted from the two-band analysis and from the SdH oscillations for ZnO-encapsulated Bi_2_Se_3_ nanoribbons on h-BN and SiO_2_ substrates, and comparison with the literature data (refs. [14,15,17]).

		Surfaces (Band 1)		Bulk (Band 2)		Top Surface *	Bulk *
**ZnO/Bi_2_Se_3_ NR on h-BN:**	***t_NR_*** **, nm**	$n_{1}$	$\mu_{1}$	$n_{2}$	$\mu_{2}$	$n_{2D,\;SdH}$	$n_{3D,\;SdH}$
A3t	29	6.43 × 10^12^	3540	4.74 × 10^12^/1.64 × 10^18^	930		
A1b	35	7.18 × 10^12^	4700	5.31 × 10^12^/1.52 × 10^18^	2052	2.40 × 10^12^	1.44 × 10^18^
D3b	34	6.24 × 10^12^	4800	4.99 × 10^12^/1.46 × 10^18^	1350		
Bi_2_Se_3_ NR on SiO_2_, sample E5 [14]	30	15.0 × 10^12^ **				2.40 × 10^12^	
Bi_2_Se_3_ NR on SiO_2_, sample BR3-10R2 [14]	63	-				2.50 × 10^12^	1.70 × 10^18^
Bi_2_Se_3_ NR on SiO_2_, sample E [17]	79	13.0 × 10^12^ *				2.90 × 10^12^	6.60 × 10^17^
Bi_2_Se_3_ NR on STO, sample B51-10 [15]	9	5.55 × 10^12^ **	1232				

* Extracted from analysis of the SdH oscillations. ** These values account only carrier density of the nanoribbon bottom surface/substrate interface.

## Data Availability

The data presented are available on request from the corresponding author.

## Supplementary Materials

The following supporting information can be downloaded at: https://www.mdpi.com/article/10.3390/nano12050768/s1.
