# Supplementary material for: Magnetotransport Studies of Encapsulated Topological Insulator Bi2Se3 Nanoribbons
Source: Nanomaterials (Basel). 2022 Feb 24;12(5):768. doi: 10.3390/nano12050768 (PMC8912099; doi:10.3390/nano12050768)
Supplement: Supplementary file 1 [file nanomaterials-12-00768-s001.zip › nanomaterials-1594021-supplementary.pdf]

nanomaterials

Supplementary Information:

Magnetotransport studies of encapsulated topological insulator Bi_2Se_3 nanoribbons

G. Kunakova[1], E. Kauranens[1], K. Niherysh[1,2], M. Bechelany[3], K. Smits[4], G. Mozolevskis[4] , T. Bauch[5], F. Lombardi[5], D. Erts[1]

[1] Institute of Chemical Physics, University of Latvia, 19 Raina Blvd., LV-1586 Riga, Latvia
[2]Research and Development department, Integrated Micro- and Nanosystems, Belarusian State University of Informatics and Radioelectronics, P. Brovki Str. 6, 220013 Minsk, Belarus
[3] Institut Européen des Membranes, IEM, UMR 5635, University of Montpellier, ENSCM, CNRS, 34095, Montpellier, France
[4] Institute of Solid State Physics, University of Latvia, Kengaraga 8, Riga, LV-1063, Latvia
[5] Quantum Device Physics Laboratory, Department of Microtechnology and Nanoscience, Chalmers University of Technology, SE-41296 Goteborg, Sweden

Citation: Kunakova, G.; Kauranens, E.; Niherysh, K.; Bechelany, M.; Smits, K.; Mozolevskis, G.; Bauch, T.; Lombardi, F.; Erts, D. Magnetotransport studies of encapsulated topological insulator Bi_2Se_3 nanoribbons. *Nanomaterials* **2022**, *12*, 768. https://doi.org/10.3390/nano12050768

Academic Editor: Yanquan Geng

Received: date: 27 January 2022
Accepted: date: 20 February 2022
Published: 24 February 2022

Publisher's Note: MDPI stays neutral with regard to jurisdictional claims in published maps and institutional affiliations.

Copyright: © 2022 by the authors. Licensee MDPI, Basel, Switzerland. This article is an open access article distributed under the terms and conditions of the Creative Commons Attribution (CC BY) license (https://creativecommons.org/licenses/by/4.0/).

1. Physical parameters of as-grown and ZnO encapsulated Bi_2Se_3 nanoribbons

Table S1. Physical parameters of as grown Bi_2Se_3 and ZnO encapsulated Bi_2Se_3 nanoribbons on Si/SiO_2 and h-BN substrates; t_{NR} is the thickness; w is the width and L is the length of the nanoribbon. $t_{h\text{-}BN}$ is the thickness of the h-BN flake.

NR batch KN_211220	NR	$t_{h\text{-}BN}$, nm	t_{NR},[1] nm	w, nm	L, µm	$n_{2D\ LF}$, $\times 10^{13}$ cm^{-2}	$n_{2D\ HF}$, $\times 10^{13}$ cm^{-2}	R_{sh}, Ω/sq.	μ,[2] $cm^2/V\cdot s$	μ_{FE}, $cm^2/V\cdot s$
+ 2 nm of ZnO	A1t[3]		44.5	804	3.60	0.89	1.21	181	2865	
			44.5	804	3.60	1.38	1.93		1793	
	A1b[3]	56	35	553	3.46	0.92	1.06	143	4100	1182
		56	35	553	3.46	1.13	1.31		3339	
	A3t[3]	32	29	1932	3.99	0.89	1.19	224	2325	2278
		32	29	1932	3.99	0.92	1.09		2547	
	D3b	43	34	368	3.47	0.87	1.18	538	981	
	D4t[3]		35	923	6.47	1.53	2.21	129	2189	501
			35	923	6.47	1.53	2.19		2213	
	E2b		32	1504	4.72	0.99	1.35	338	1368	
As grown	A4t		28	409	5.61	2.52	2.72	342	672	
	B1[3]		40	1688	5.39	0.96	1.16	263	2040	
			40	1688	5.39	0.88	1.04		2276	
	B2		31	1436	4.60	0.95	1.19	232	2257	
	C2b[3]		38	1104	5.47	1.07	1.36	261	1754	
			38	1104	5.47	1.64	1.70		1409	
	C5b		30	640	4.73	0.72	1.33	224	2093	

[1] Thickness accounts for the two ~2 nm thick ZnO layers.

[2] μ is calculated using the $n_{2D\ HF}$.

[3] Values of the carrier densities are calculated from the two datasets of $R_{xy}(B)$, where each of them is measured using different pair of the Hall electrodes, on the same nanoribbon.

Table S1 summarizes nanoribbon dimensions and charge transport parameters calculated from the magnetotransport data. Nanoribbon and exfoliated h-BN flake thicknesses are measured using atomic force microscopy, while the nanoribbon width is determined via scanning electron microscopy imaging. Values of the charge carrier densities $n_{2D\ LF}$ and $n_{2D\ HF}$ are calculated using eq.1 of the main text, and subscript "LF" and "HF" represent range of the magnetic field used to determine the slope of the anti-symmetrized $R_{xy}(B)$ data. LF and HF correspond to the 0 – 2.5T and 7 – 9T magnetic field range, respectively. R_{sh} is the sheet resistance, calculated as $R_{xx}\ w/L$, where R_{xx} is the longitudinal resistance measured in a four probe configuration, at B = 0T and T = 2K. Calculated R_{sh} values for as-grown and ZnO/Bi_2Se_3 nanoribbons are similar, about ~ 200 Ω/sq. Hall mobility μ is calculated as $1/(R_{sh} e\ n_{2D,HF})$. For both as-grown and encapsulated nanoribbons the values of μ are also very close, about 2000 and ~2200 cm^2/Vs respectively.

The field effect mobility μ_{FE} can be evaluated as $\mu_{FE} = \frac{L}{w} \cdot \frac{dG_{xx}}{dV_g} \cdot \frac{1}{C}$; where G_{xx} is the conductance in the linear region of $G_{xx}(V_g)$ dependence, C is the back-gate capacitance, w and L is the width and length of the nanoribbon. The back-gate capacitance can be estimated as $C = \varepsilon\varepsilon_0/d$, where ε_0 is the vacuum permittivity, ε is the dielectric constant of the SiO_2 or h-BN, d is the thickness of the back-gate dielectric (ε_{SiO2}~3.9, ε_{hBN}~4).

2. Magnetotransport data analysis using the two-carrier model

Figure 1S a, c shows the conductance tensor element $G_{xy}(B)$ and $G_{xx}(B)$ curves calculated in agreement with the equations 2.a and 2.b of the main text with the best fits from the two-carrier model (equations 3.a and 3.b, main text), for the ZnO/Bi_2Se_3 nanoribbons

A1b and D3b. Extracted values of the charge carrier densities at V_g = 0V for the nanoribbon A1b are $\boldsymbol{n_1}$ = 7.18·10[12] cm[−2] and $\boldsymbol{n_2}$ = 5.31·10[12] cm[−2] and mobilities $\boldsymbol{\mu_1}$ = 4700 cm[2]/Vs, $\boldsymbol{\mu_2}$ = 2052 cm[2]/Vs. These values of the two bands are similar also for the nanoribbon D3b, where $\boldsymbol{n_1}$ = 6.24·10[12] cm[−2]; $\boldsymbol{n_2}$ = 4.99·10[12] cm[−2] and $\boldsymbol{\mu_1}$ = 4800 cm[2]/Vs; $\boldsymbol{\mu_2}$ = 1350 cm[2]/Vs. The band 1 characterised with the highest $\boldsymbol{n_1}$ and $\boldsymbol{\mu_1}$ values representing the carriers from the nanoribbon surfaces, while the band 2 with carrier density $\boldsymbol{n_2}$ and mobility $\boldsymbol{\mu_2}$ correspond the nanoribbon bulk. The back gate voltage dependences of $\boldsymbol{n_1}$; $\boldsymbol{n_2}$ and $\boldsymbol{\mu_1}$; $\boldsymbol{\mu_2}$ for nanoribbon A1b are plotted in Figure 1S b. The $\boldsymbol{n}$ values of both bands slightly decrease applying negative back-gate voltages, indicating moderate depletion of the interface and the bulk carriers. The $\boldsymbol{\mu_1}$; $\boldsymbol{\mu_2}$ values remain instead practically unchanged in the back-gate voltage range 0 - –40V. At larger negative back-gate voltage of –65V the $\boldsymbol{\mu_2}$ representing the bottom surface / substrate interface carriers increase to about 2700 cm[-2]/Vs, indicating reduction of the scattering centres at the bulk.

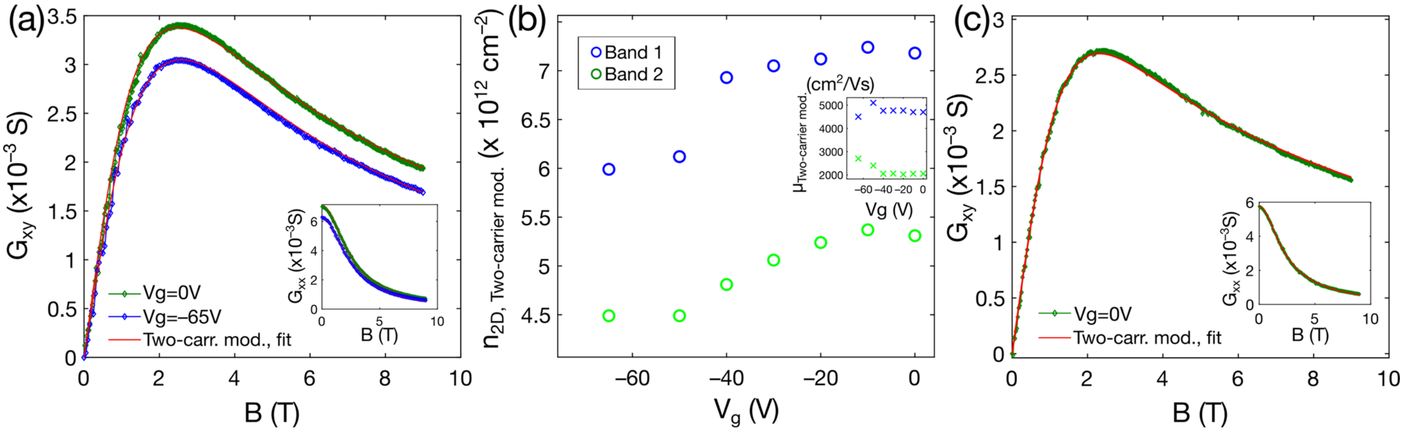

Figure S1. Two-carrier analysis of the ZnO/Bi_2Se_3 nanoribbon magnetotransport data measured on nanoribbon A1b: (**a**) Conductance tensor element $G_{xy}(B)$ curves at V_g = 0V, and V_g = –65V, with the fits of the two-carrier model (red solid lines), and in the inset - corresponding fitted $G_{xx}(B)$ data. (**b**) From the two-carrier mod. extracted values of the carrier densities n_1; n_2, and mobilities μ_1; μ_2 (in the inset), plotted as a function of the back-gate voltage. Nanoribbon D3b: (**c**) Conductance tensor element $G_{xy}(B)$ and $G_{xx}(B)$ curves at V_g=0V, red solid lines are the fits of the two-carrier model.

3. Shubnikov-de Haas oscillations of the ZnO encapsulated Bi_2Se_3 nanoribbons

The magnetic field dependence of the longitudinal resistance R_{xx} for a ~35 nm thin ZnO/Bi_2Se_3 nanoribbon shows oscillations in R_{xx}, at magnetic field above ~5 T. These are the Shubnikov-de Hass oscillations, and the extracted oscillatory part ΔR_{xx} as a function of the inverse magnetic field is depicted in Figure 2S b. The FFT spectra of these data shows two main frequencies F_1 ~ 40 T and F_2 ~ 99 T. The highest frequency F_2 has previously been reported to correspond to the carriers from the surface Dirac states, originating from the nanoribbon top surface [1]. In this case, the 2D carrier density $n_{2D,SdH}$ can be estimated as $k_F^2/4\pi$; $F = (\hbar/(2\pi e))A_0$; $A_0 = \pi k_F^2$, where k_F is the Fermi wave vector, F is the SdH oscillation frequency, A_0 is the Fermi surface area. From F_2 we calculate $n_{2D,SdH}$= 2.40·10[12] cm[−2]. This leaves an open question for the identification of the lowest frequency F_1. F_1 can be attributed to the bulk carriers which results in a 3D carrier density $n_{3D,SdH,\ Bulk}$ = $1/(2\pi)^2(4/3)k_F^3$ is 1.4·10[18] cm[−3] in agreement with values obtained from Hall measurements (see 2 band analysis in main text).

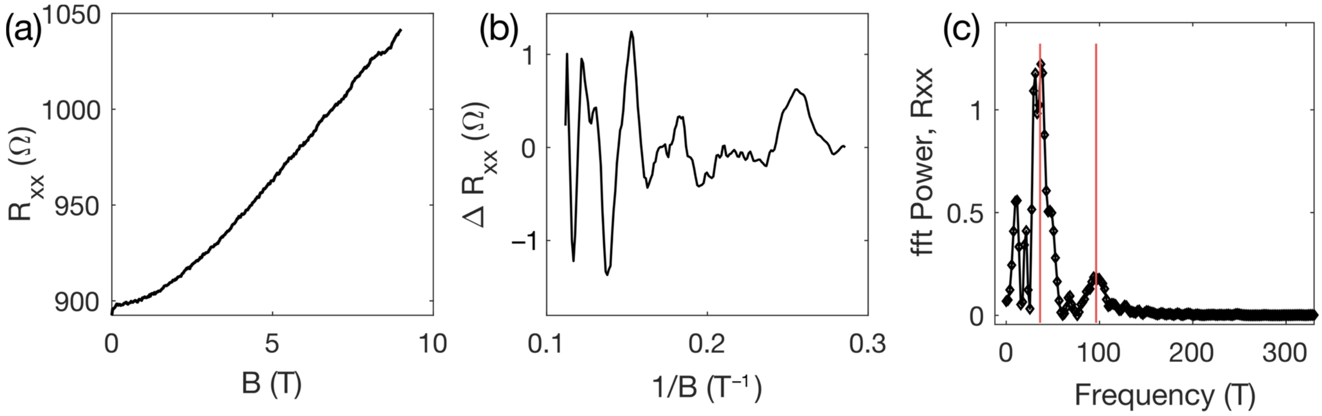

Figure S2. (**a**) $R_{xx}(B)$ dependence. (**b**) Oscillatory part of the $R_{xx}(B)$, plotted versus inverse magnetic field. (**c**) FFT spectra of the $R_{xx}(B)$ data. Data shown correspond to ZnO/Bi_2Se_3 nanoribbon A1b.

References

[1] G. Kunakova, L. Galletti, S. Charpentier, J. Andzane, D. Erts, F. Léonard, C. D. Spataru, T. Bauch, and F. Lombardi, *Bulk-Free Topological Insulator Bi2Se3 Nanoribbons with Magnetotransport Signatures of Dirac Surface States*, Nanoscale **10**, 19595 (2018).
